# Preventing Long COVID With Metformin

**DOI:** 10.1093/cid/ciaf700

**Published:** 2026-01-29

**Authors:** Carolyn T Bramante, David R Boulware

**Affiliations:** Division of General Internal Medicine, Department of Medicine, University of Minnesota Medical School, Minneapolis, Minnesota, USA; Division of Infectious Diseases and International Medicine, Department of Medicine, University of Minnesota Medical School, Minneapolis, Minnesota, USA

**Keywords:** long COVID prevention, metformin, SARS-CoV-2

**(See the Major Article by Chaichana et al on pages e423–32.)**

Chaichana et al conducted a sequential trial emulation to assess whether starting metformin after severe acute respiratory syndrome coronavirus 2 (SARS-CoV-2) infection prevented the development of post-coronavirus 19 (COVID 19) condition or long COVID [1]. Their work provides critical validation of previously published randomized clinical trial data and is consistent with emerging data. We review this body of literature and why metformin should now be offered to outpatient adults for treating acute SARS-CoV-2 infection to prevent long COVID [2].

Repurposing a chronic diabetes medication for acute viral infection may seem counterintuitive, so we will briefly review the rationale. Early papers on biguanides were for use against viruses and malaria [3–5]. In about 1940, biguanides fell out of favor because of lactic acidosis with phenformin and buformin [3]. Metformin was less potent for lowering glucose but had fewer safety concerns and was approved for treating type 2 diabetes [3]. This led to studies of metformin's anti-inflammatory actions and cohort studies in which there was no increased risk of lactic acidosis with metformin [6–9].

In the 2000's, metformin was studied in vitro against viruses because of its host-directed, immunometabolic actions [10]. In 2020, observational, in silico computer modeling and in vitro studies of SARS-CoV-2 added to the rationale for studying metformin as acute SARS-CoV-2 treatment [10–13]. Given these multiple streams of evidence, metformin's low cost, wide availability, tolerability, and safety with no need for monitoring during short-term use, it was important to test metformin versus placebo for outpatient treatment of SARS-CoV-2 [14].

In July 2021, we added a long-term clinical outcome to the COVID-OUT randomized trial protocol and consent to assess whether treatment during acute infection prevented long-term sequelae. At that time, it was unclear which specific symptoms constituted long-term sequelae, or what cutoffs to use for each symptom's duration, severity, or frequency. Symptom-based outcomes could also not be verified in electronic health records, and we wanted an outcome that could be verified in other sources of data.

Thus, to best capture the definition of long COVID in clinical practice, and to still capture the outcome if that definition evolved during the course of the trial, we decided to ask participants: “Have you been told by a medical provider that you have long COVID?” This way of ascertaining long COVID allowed us to obtain the source medical records to confirm the participant-reported diagnosis. These long COVID diagnoses were made by clinicians in the community who were blinded, not involved in the study, and who were using the resources available for diagnosing this new disease while simultaneously ruling out other issues.

In the COVID-OUT trial, the metformin group had a 41% lower risk of long COVID over 10 months of follow-up [15]. We need to clarify the first line of the abstract by Chaichana et al—the authors state “A subgroup analysis of the COVID-OUT trial's long-term outcome found that starting metformin within 3 days of COVID-19 reduced PCC incidence by 63%.” This could imply that the effect was observed in only a subgroup and not in the full sample. However, the 41% lower risk was for the full sample, hazard ratio 0.59 (95% confidence interval [CI] .39–.89). The 63% lower risk they quoted, hazard ratio 0.37 (95% CI .15–.95), was among the subgroup who started study drug within 3 days of COVID-19 symptom onset.

A small mechanistic randomized trial testing metformin versus placebo on viral load found metformin reduced SARS-CoV-2 by 93.2%, compared to 78.3% with placebo (*P* = .013); and the time to an undetectable viral load was 3.3 days for metformin and 5.6 days for placebo (*P* = .043) [16]. Metformin also reduced viral load relative to placebo (−0.56 log_10_ copies/mL) in COVID-OUT [17].

Given these findings, another randomized trial was necessary to confirm that metformin prevents long COVID and in the evolving pandemic. Thus, the ACTIV-6 trial tested metformin versus placebo. COVID-OUT had excluded those with a known prior infection and had required a minimum body mass index (BMI) of 25 kg/m^2^ or greater [18]. Because there were no safety concerns for starting metformin during acute infection in the COVID-OUT trial, ACTIV-6 took the important step in understanding the safety of metformin during acute infection including in those with a normal BMI and those with prior infection.

ACTIV-6 used the same dose as COVID-OUT: Metformin hydrochloride, 500 mg immediate release tablets: 500 mg for 1 day; 500 mg twice per day for 4 days; 500 mg in the morning and 1000 mg in the evening for 9 days (36 tablets total). Metformin did not cause clinically significant gastrointestinal side effects and there were no safety concerns in either trial [14, 19]. In ACTIV-6, there were 2 episodes of participant-reported hypoglycemia in the metformin group (0.14%), and 4 in the placebo group (0.26%).

**Figure 1:**
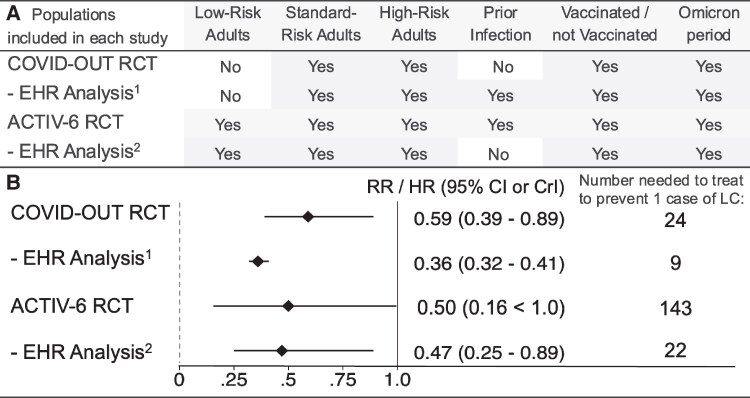
Overview of 4 studies (2 randomized clinical trials and 2 electronic health record analyses in similar populations) that tested whether metformin during acute severe acute respiratory syndrome conronavirus 2 (SARS-CoV-2) infection prevents long COVID. *A*, This table shows the population included in each study. The ACTIV-6 RCT and its confirmatory EHR analysis both included low-, standard-, and high-risk adults. The COVID-OUT trial and its confirmatory EHR analysis included standard- and high-risk adults. All studies included vaccinated and nonvaccinated individuals, and individuals infected after the start of the Omicron variant. Two of the studies included persons with a known prior infection. Both RCT's allowed pregnant or lactating individuals to enroll and be randomized to metformin or placebo. The ACTIV-6 RCT and its confirmatory analysis included individuals prescribed nirmatrelvir-ritonavir during acute infection. *B*, This is a forest plot with the point estimate and 95% confidence interval (CI) or 95% credible interval (CrI) for the 4 studies. Values < 1.0 indicate benefit from metformin. The right column shows the number needed to treat (NNT) in each study to prevent one diagnosis of LC. NNT is calculated by dividing 1.0 by the absolute risk reduction. The absolute risk reduction is calculated by subtracting the percent of participants in the metformin group with the outcome from the percent of participants in the placebo group with the outcome. ^1^Chaichana et al 2025; ^2^ Bramante et al 2025. Abbreviations: LC, long COVID; EHR, electronic health record; RCT, randomized clinical trial; RR, risk ratio; HR, risk hazard ratio.

In ACTIV-6, the primary outcome was ascertained by asking participants: “Please choose the response that best describes the severity of your COVID-19 symptoms today” on Day 180. A secondary outcome on Day 180 was the question used in COVID-OUT: “Have you been told by a medical provider that you have long COVID since your last survey?” The results were similar to COVID-OUT, risk ratio 0.50 (95% credible interval .16 < 1.0) [20]. A second target trial emulation (electronic health record study) confirmed the ACTIV-6 results in adults with a normal BMI or greater, risk ratio of 0.47 (95% CI, .25–.89), Figure 1 [21].

Two additional methodologic points about these studies. First, clinician-diagnosed long COVID was a secondary outcome in both randomized clinical trials, and secondary outcomes can be used for making clinical treatment guidelines [22, 23]. Second, the patient and provider communities still struggle with diagnosing long COVID. The purpose of randomized trials is not to identify the true incidence of a disease in the general population, but rather to obtain an unconfounded comparison between starting medication or starting placebo. Some patients suffering with long COVID may not receive a diagnosis from a medical provider for a variety of factors. These measured and unmeasured factors could confound an estimation of disease prevalence in the community, but they do not confound the comparison of medication versus placebo because they are distributed by randomization. Target trial analyses are not randomized and may have confounding, but their purpose is to assess consistency of effect on a clinical diagnosis.

In summary, 2 large, randomized, placebo-controlled clinical trials in highly relevant populations show that starting metformin at the time of acute infection with SARS-CoV-2 is safe and lowers the risk of developing long COVID. Both trials enrolled after the Omicron variant appeared, enrolled adults with prior immunity, and have been validated in target trial analyses of similar populations. The studies included adults considered to be low, standard, and high-risk. There are no drug–drug interactions that prevent someone from taking both metformin and therapies currently listed in guidelines. Adding metformin to clinical treatment guidelines for nonhospitalized adults with acute SARS-CoV-2 infection would likely help disseminate the data to more individuals who match the populations in these studies (Figure 1).

Repurposing medications, even between infectious versus noninfectious indications, dates back to at least the 1950's, when iproniazid (a xenobiotic used to treat tuberculosis), was studied for treating depression [24]. This repurposing of an antimicrobial to treat a chronic daily disease is considered the origin of modern psychopharmacology. William Osler also referenced repurposing: “The young physician starts life with 20 drugs for each disease, and the old physician ends life with one drug for 20 diseases” [25].
